# *In vitro* decidualisation of canine uterine stromal cells

**DOI:** 10.1186/s12958-015-0066-4

**Published:** 2015-08-05

**Authors:** Ewa Kautz, Paula de Carvalho Papa, Iris M. Reichler, Aykut Gram, Alois Boos, Mariusz P. Kowalewski

**Affiliations:** Institute of Veterinary Anatomy, Vetsuisse Faculty, University of Zurich, Winterthurerstrasse 260, CH-8057 Zurich, Switzerland; Department of Surgery, Faculty of Veterinary Medicine and Animal Sciences, Sector of Anatomy, University of Sao Paulo, Sao Paulo, Brazil; Section of Small Animal Reproduction, Clinic of Reproductive Medicine, Vetsuisse Faculty, University of Zurich, Zurich, Switzerland

**Keywords:** Domestic dog, Decidualisation, *in vitro* model

## Abstract

**Background:**

The uterine response to the presence of embryos is poorly understood in the domestic dog (Canis familiaris). The intimate embryo-maternal cross-talk, which begins following the hatching of blastocysts and embryo attachment leads to strong structural and functional remodelling of the uterus. A part of this process is decidualisation, comprising morphological and biochemical changes that result in formation of maternal stroma-derived decidual cells. These are an integral part of the canine *placenta materna*, which together with the maternal vascular endothelium are the only cells of the canine endotheliochorial placenta able to resist trophoblast invasion. These cells are also the only ones within the canine placenta expressing the progesterone receptor (PGR). Understanding the decidualisation process thus appears essential for understanding canine reproductive physiology.

**Methods:**

Here, we investigated the capability of canine uterine stromal cells to decidualise *in vitro*, thereby serving as a canine model of decidualisation. A dbcAMP-mediated approach was chosen during a time course of 24 - 72 h. Tissue material from six (*n* = 6) healthy, dioestric bitches was used (approximately 2 weeks after ovulation). Cells were characterized by differential staining, nearly 100 % of which were vimentin-positive. Scanning and transmission electron microscope analyses were applied, and morphological changes were recorded with a live cell imaging microscope. Expression of several decidualisation markers was investigated.

**Results:**

The *in vitro* cultured stromal cells acquired characteristics of decidual cells when incubated with 0.5 mM dbcAMP for 72 h. Their shape changed from elongated to rounded, while ultrastructural analysis revealed higher numbers of mitochondria and secretory follicles, and an increased proliferation rate. Elevated expression levels of IGF1, IGF2, PRLR and ERα were observed in decidualised cells; PRL and ERβ remained mostly below the detection limit, while PGR remained unaffected. The expression of smooth muscle α actin (αSMA), another decidualisation marker, was strongly induced. Among prostaglandin system members, levels of COX2 (PTGS2) and of PGE2-synthase (PTGES) were upregulated. Expression of the PGE2 receptors, PTGER2 and PTGER4, was clearly detectable.

**Conclusion:**

An *in vitro* decidualisation model with canine uterine stromal cells was successfully established, allowing future, more detailed studies to be undertaken on the underlying molecular and endocrine mechanisms of canine decidualisation.

**Electronic supplementary material:**

The online version of this article (doi:10.1186/s12958-015-0066-4) contains supplementary material, which is available to authorized users.

## Introduction

Successful establishment of pregnancy requires highly orchestrated interactions between embryonic and maternal uterine tissues, which undergo specific morphological and biochemical changes to establish the uterine milieu required for proper embryo development. In animal species exhibiting invasive types of placentation, *e.g*., higher order primates and rodents which develop hemochorial placentas, or dogs and cats which exhibit endotheliochorial placentas, these restructuring changes are very intense and result in decidua formation. Reprogramming of the endometrial stromal compartment during decidualisation involves transformation of stromal cells into decidual cells, which are involved in coordinating the processes of embryo implantation, placenta formation and development of the conceptus [1, 2]. This specific tissue differentiation remains under the control of progesterone (P4) and oestrogens [3, 4]. Problems during decidualisation are frequent causes of implantation failures in humans [5], and may contribute to abnormal placentation, sometimes resulting in severe conditions such as formation of *Placenta accreta* which is characterised by excessive trophoblast invasion into the decidua [6]. In other species, such as dogs, the latter anomaly, i.e. exaggerated trophoblast invasion, can result in a condition known as subinvolution of placental sites (SIPS) [7].

In the domestic dog, the establishment and maintenance of pregnancy depends entirely on P4 secreted from Corpora lutea (CL) because there is no placental steroidogenesis in this species [8–10]. Furthermore, in the absence of pregnancy, the dog also lacks an endogenous luteolysin, which results in a similar luteal life span and circulating hormone profiles during pregnancy and in pseudopregnant bitches [11]. Displaying these unique features, reproductive function in the dog differs distinctly from other domestic animal species. Consequently, being devoid of an embryo-derived anti-luteolytic principle found in livestock, *e.g*., cattle or pigs [12–14], the dog appears to be an interesting model for investigating alternative mechanisms involved in the establishment of pregnancy in mammals. However, there is only little available information concerning the uterine milieu prior to and during implantation and placentation in dogs. Just recently [15], the effects exerted by free-floating embryos (days 10–12 after fertilisation) on the canine pre-implantation uterus were investigated, revealing increased expression of several decidualisation markers, *e.g*., insulin-like growth factor (IGF) 2, prolactin receptor (PRLR) and oestrogen receptor α (ERα, ESR1). Besides being clearly detectable in the uterine epithelial compartments, their expression was also detected in stromal cells. Interestingly, in contrast to rodents or primates [16, 17], the capacity of the canine uterus to produce PRL was found to be very low [15]. Among the prostaglandin (PG) family members, especially the expression of PGE2-synthase (PTGES) and one of the respective PGE2 receptors, PTGER2, was significantly upregulated in response to the presence of free-floating embryos [15].

The embryo-induced uterine functional differentiation in the dog becomes very intense following implantation, *i.e.*, by days 17–18 after fertilisation and placentation [18, 19]. The latter event is characterised by strong, trophoblast-induced structural and functional remodelling of the endometrium, eventually leading to the formation of highly specialised canine decidual cells. Together with maternal vascular endothelial cells, canine decidual cells are able to resist the strong proteolytic activity of embryonic cells. Thus, their role in modulating trophoblast invasiveness, and thereby protecting maternal tissues from excessive degradation, can be assumed. Most importantly, these are also the only cells of the dog placenta expressing PGR and ERα (ESR1) and, thereby, must play a pivotal role in the maintenance of pregnancy [20, 21]. Interfering with their function, rather than their expression, *e.g*., by applying a selective PGR blocker, will unequivocally lead to pre-term parturition/abortion by activating utero-placental PG synthesis [20]. Acting at the level of decidual cell-targeted expression of its receptor (OTR, OXTR), oxytocin appears to be an important mediator in this process [22]. Nevertheless, the underlying endocrine mechanisms associated with the decidualisation process in the dog remain unknown.

*In vitro* decidualisation can be induced by several stimuli. One of these, cyclic AMP (cAMP), is not only an important mediator of P4-induced decidualisation [23], but acts as a stronger stimulus than P4 in inducing expression of decidualisation markers in endometrial stromal cells [24–26]. Here, aiming to understand decidualisation in the dog, the capability of canine uterine stromal cells to undergo this process *in vitro* was investigated. Cells were isolated from uteri of naturally oestrogenized, dioestrous dogs. Due to our limited access to the experimental material, and bearing in mind the above-mentioned strong decidualisation potential of cAMP, a cAMP-based protocol was applied. Then, specific morphological, ultrastructural and functional phenomena associated with the *in vitro* decidualisation process were identified and evaluated.

## Materials and methods

### Collection of tissues and isolation of cells

Uterine tissues were used from six (*n* = 6) non-pregnant, clinically healthy bitches, submitted for routine ovariohysterectomy at the Section of Small Animal Reproduction, Clinic of Reproductive Medicine, Vetsuisse Faculty, University of Zurich, Zurich, Switzerland. Surgeries were performed during early non-pregnant dioestrus, approximately 12–14 days after clinical signs of heat had ceased. Immediately after collection, uteri were washed with sterile phosphate-buffered saline (PBS) solution and separated from surrounding connective tissues. Uteri were slit longitudinally and the endometrial layers were mechanically separated. After washing with PBS, tissue fragments were enzymatically dissociated by stirring for 2 h at 37 °C in PBS containing 0.15 % collagenase (Sigma-Aldrich Chemie GmbH, Buchs, CH). Subsequent removal of undissociated tissue fragments was achieved by filtering through a 75 μm nylon strainer (BD Biosciences, Basel, CH). Following this, cells were washed three times in PBS with centrifugation steps in between (400 × g, 10 min, 4 °C). Afterwards, pellets were resuspended with culture medium (DMEM High Glucose, pH 7.2-7.4, with 10 % heat inactivated FBS, containing 100 U/ml penicillin and 100 μg/ml streptomycin, and 1 % ITS (Insulin-Transferrin-Selenium); all from Chemie Brunschwig AG, Basel, CH) and seeded into 150 ml culture flasks (Corning, Amsterdam, NL). Following 1 h of incubation in a humidified incubator at 37 °C under 5 % CO_2_ in air, needed for adhesion of fibroblast stromal cells and their separation from epithelial cells (differential adhesion time), the medium was replaced with fresh cell-free medium. The attached stromal cells were further cultured under the same conditions until confluence, which was reached after approximately 4–5 days. Cells after the first passage were used for further experiments. Then, after trypsinisation and harvesting, cells were seeded into 6-well plates at different concentrations: 4 × 10^5^ per well for 24 h and 2 × 10^5^ per well for 48 h and 72 h incubation, respectively. When seeded at less than 2 × 10^5^ per well, cells easily attached but exhibited retarded growth, most likely due to the low density and lack of sufficient cell-to-cell contact. On the other hand, when cultured longer than 72 h, stimulated cells became over-confluent. For immunocytochemistry, sterile glass cover slips were placed into the wells to allow the seeded cells to adhere to them. Following seeding, cells were further cultured under the above conditions for approximately 24 h and then used for experiments. Prior to stimulation, cells were washed with PBS, thereby removing the serum-containing medium. Subsequently, cells were treated with N6,2-O-Dibutyryladenosine-3,5-cyclic monophosphate (dbcAMP) in stimulation medium (DMEM High Glucose, pH = 7.2-7.4; penicillin/streptomycin as above), containing 0.1 % bovine serum albumin (BSA), in the following concentrations: 0 mM dbcAMP for the non-treated control, or 0.1 mM, 0.3 mM or 0.5 mM dbcAMP. In pilot experiments, serum-free medium was used for stimulation, which resulted in detachment of cells within 2 h after stimulation. Inclusion of BSA at the indicated concentration of 0.1 % proved to be the lowest threshold determined empirically in experiments comprising serial dilution of BSA, needed for cells to prevent their detachment (data not shown).

### Live cell imaging microscopy

Cells seeded in 6-well plates were placed on a LeicaDMI 6000B fluorescence microscope equipped with a Leica DFC360FX camera and an automated stage for live cell imaging (allowing for simultaneous observation of cells undergoing *in vitro* decidualisation and comparison with non-treated controls), enclosed within a thermostatically-controlled cabinet. Stimulations for live cell imaging microscopy were done in duplicate as follows: non-treated controls and 0.5 mM dbcAMP. The focal planes were set for image capture, and images were taken with a 10x objective every 5 min for 72 h, resulting in a total of 864 cycles. Culture conditions were: 20 % O_2_, 5 % CO_2_, at 37 °C. The resulting time-lapse videos were displayed at a fast speed to provide an accelerated view of *in vitro* decidualisation of canine stromal cells. Representative videos of control and *in vitro* decidualised canine stromal cells are presented in the supplemental material. The density of cells was quantified at cycles 144 (12 h), 288 (24 h), 576 (48 h) and 864 (72 h) and was evaluated relative to the surface of the applied fields of view using the MCID Analysis Software (InterFocus Limited, Linton, UK). *N*-fold changes in relative target area were determined by applying one-way analysis of variance (ANOVA) followed by Dunnett’s Multiple Comparison test. Student’s *t*-test was applied to test the effect of treatment on cell density at the selected time points. Statistical tests were performed using the GraphPad 3.06 program (GraphPad Statistical Software, San Diego, CA, USA). Results are presented as *n*-fold changes in the relative target area, reflecting the density of cells as % of the surface.

### Immunocytochemistry and immunohistochemistry

Immunofluorescence was used for staining canine uterine primary cells seeded in 6-well plates on sterile cover slips. Cells were fixed by adding formaldehyde to cell cultures at a final concentration of 2 % for 10 min at 37 °C. After fixation, each well was washed twice with ice-cold PBS and submitted to the staining procedure described previously [27]. Briefly: cover slips were washed twice with ice-cold PBST (PBS/0.25 % Triton X) followed by antigen retrieval in 50 mM glycine (Sigma-Aldrich) in PBS for 5 min, and two washing steps in PBST (5 min each). A 1 % solution of goat serum diluted in PBST was used for blocking non-specific binding sites (30 min). Next, primary antibodies were applied for 2 h. These were: monoclonal mouse anti-vimentin (dilution 1:100), affinity-purified polyclonal rabbit anti-cytokeratin (dilution 1:300) and monoclonal mouse anti-human smooth muscle alpha actin (αSMA) (dilution 1:100), all from from Dako North America, Inc., CA, USA. Negative controls included: samples omitting primary antibody, and those where both primary and secondary antibodies were omitted (for the autofluorescence control). Fluoresceinated goat anti-rabbit IgG antibody FI-1000 (Vector Laboratories, Burlingame, CA, USA) and fluoresceinated goat anti-mouse IgG antibody FI-2000, were used as secondary antibodies and applied in PBST for 1 h. Nuclear staining was achieved by adding 4′,6-diamidino-2-phenylindole (DAPI) (Sigma-Aldrich) to the secondary antibody solution. Cells were post-fixed with 2 % formaldehyde. Glycergel® (Dako North America) was used for mounting cover slips on microscope slides.

Uterine cross-sections 2-3 μm thick from early pregnant bitches, pre-implantation stages of pregnancy (days 10–12 after fertilisation; n = 3) and utero-placental compartments from mid-pregnant dogs (35–40 days of pregnancy; n = 3), were immunhistochemically stained for αSMA with our standard immunoperoxidase method [28, 29]. The primary antibody was the same as for the immunocytochemistry and used at 1:100 dilution. The secondary antibody was horse anti-mouse IgG BA-2000 (dilution 1:100; Vector Laboratories). The Vectastain ABC kit (Vector Laboratories) was used for enhancing signals. Peroxidase detection was done with the Liquid DAB+ substrate kit (Dako Schweiz AG, Baar, CH). Negative controls included: omitting the primary antibody, and an isotype control, rabbit IgG irrelevant antibodies I-1000 from Vector Laboratories used at the same concentration as the primary antibody (not shown).

### Electron microscopy analysis

Previously described protocols for conventional transmission electron microscopy (TEM) and scanning electron microscopy (SEM) were used [30]. Briefly: for TEM, cells were seeded in a 6-well plate onto sapphire discs and grown for 72 h with/without 0.5 mM dbcAMP. After treatment, cells were fixed with 2.5 % glutaraldehyde in 0.1 M Na/K-phosphate, pH 7.4 for 1 h at 4 °C, and kept in 0.1 M Na/K-phosphate overnight at 4 °C. Then, samples were post-fixed with 1 % osmium tetroxide (OsO_4_) in 0.1 M Na/K-phosphate for 1 h at 4 °C. Afterwards, dehydration was performed in a graded series of ethanol starting at 70 % followed by two changes in acetone and embedding in Epon. Sections 60–80 nm thick were stained with uranyl acetate and lead citrate and analysed in a transmission electron microscope (CM12, Philips, Eindhoven, The Netherlands) equipped with a CCD camera (Ultrascan 1000, Gatan Pleasanton, CA, USA) at an acceleration voltage of 100 kV.

For SEM, cells were grown on cover slips 10 mm in diameter (Mattek, Ashland, MA, USA). Following stimulation under the conditions described above, 2.5 % glutaraldehyde in 0.1 M Na/K-phosphate, pH 7.4, was added to the warm culture medium. Then dishes were kept at 4 °C for 1 h, the medium was replaced by 0.1 M Na/K-phosphate, and stored at 4 °C overnight. After post-fixation with 1 % osmium tetroxide in 0.1 M Na/K-phosphate, pH 7.4, at 4 °C, cells were dehydrated in a graded series of ethanol starting at 10 %, then critical-point dried (BAL-TEC, CPD 030, Balzers, Liechtenstein), coated with 4 nm platinum in a high vacuum sputtering device, (BAL-TEC SCD500), and examined in a SEM (Zeiss Supra 50 VP, Zeiss, Oberkochen, Germany) at an acceleration voltage of 5 kV using the secondary electron detector.

### RNA isolation, reverse transcription (RT), semi-quantitative (TaqMan) PCR and data evaluation

Total RNA was isolated from primary stromal cells using TRIZOL®-Reagent (Invitrogen, Carlsbad, CA) according to the manufacturer’s instructions. The RNA content was measured with a NanoDrop 2000 UV–vis Spectrophotometer® (Thermo Fisher Scientific, Reinach, CH). In order to remove potential genomic DNA contaminants resulting from the TRIZOL® protocol, RQ1 RNAse-free DNAse (Promega, Dübendorf, CH) was used following the manufacturer’s protocol. For complementary DNA (cDNA) synthesis, random hexamers were used as primers with RT reagents from Applied Biosystems, Foster City, CA, USA, following the manufacturer’s protocol and as previously published [28, 31]. Reverse transcription was carried out in an Eppendorf Mastercycler® (Vaudaux-Eppendorf AG, Basel, CH) under the following conditions: 8 min at 21 °C and 15 min at 42 °C, after which the reaction was stopped by incubation for 5 min at 99 °C. Semi-quantitative real time (TaqMan) PCR was performed in an automated fluorometer ABI PRISM® 7500 Sequence Detection System (Applied Biosystems) according to our previously described protocol [20, 32]. Fast Start Universal Probe Master (ROX®) (Roche Diagnostics AG, CH) was used with cDNA corresponding to 200 ng of DNAse-treated total RNA per sample. All reactions were run in duplicate. The following negative controls were applied: autoclaved water instead of cDNA and the so-called “RT minus” controls (samples which were not reverse transcribed), allowing exclusion of potential contaminants in reagents used for RT-PCR and confirming the accuracy of DNase-treatment, respectively. The semi-quantitation of target gene expression was carried out using three different endogenous reference genes (GAPDH, 18SrRNA and cyclophilin A). The list of primers and TaqMan Probes is presented in Table 1. The comparative CT method (ΔΔCT method) was applied according to the instructions of the ABI PRISM 7500 Sequence Detection System and as previously published [20, 32]: expression of each target gene relative to the reference genes, and normalised to the calibrator, *i.e.,* sample with the lowest amount of the respective target gene transcripts, was calculated. The average amounts of relative gene expression determined in relation to each of the reference genes for a sample were used as the normalization factor. The specificity of the selected PCR products was confirmed by sequencing (Microsynth, Balgach, CH). Canine-specific TaqMan Gene Expression Assays purchased from Applied Biosystems were used for: cyclophilin A (Prod. No. Cf03986523- gH), IGF-1 (Prod. No. Cf02627846_m1) and IGF-2 (Prod. No. Cf02647136_m1). Selected amplicons of each of the genes were sent for commercial sequencing (Microsynth). Statistical analysis was performed using the GraphPad 3.06 software. One-way analysis of variance (ANOVA) for each of the treatment groups, *i.e*. 24 h, 48 h and 72 h, was applied followed by the Tukey-Kramer Multiple Comparison Test. Numerical data are presented as the mean ± standard deviation (SD).

**Table 1:** List of primers and TaqMan Probes used for the semi-quantitative RT-PCR

Primer	Accession numbers	Primer sequence	Product length (bp)
*GAPDH*	AB028142	Forward: 5′-GCT GCC AAA TAT GAC GAC ATC A-3′	75
		Reverse: 5′-GTA GCC CAG GAT GCC TTT GAG-3′	
		TaqMan Probe: 5′-TCC CTC CGA TGC CTG CTT CAC TAC CTT-3′	
*18SrRNA*	FJ797658	Forward: 5′-GTC GCT CGC TCC TCT CCT ACT-3′	125
		Reverse: 5′-GGC TGA CCG GGT TGG TTT-3′	
		TaqMan Probe: 5′-ACA TGC CGA CGG GCG CTG AC-3′	
*IGFR1*	XM545828	Forward: 5′-GGA CGT GCC TGG CAT-3′	119
		Reverse: 5′-CAC TCT TAG CCC CAC GGA TGT-3′	
		TaqMan Probe: 5′-AGC CCT GGA CGC AGT ATG CGG-3′	
*PRLR*	HQ267784	Forward: 5′-GGA TCT TTG CCG TTC TTT-3′	92
		Reverse: 5′-AAG GAT GCA GGT CAC CAT GCT AT-3′	
		TaqMan Probe: 5′-ATT ATG GTC GTA GCA GTG GCT TTG AAA GGC-3′	
*PRL*	NM_001008275	Forward: 5′-CAAGCC CAA CAG ATC CAC CAT-3′	104
		Reverse: 5′-ATC CCC CGC ACT TCT GTG A-3′	
		TaqMan Probe: 5′-CTG AGG GTG CTG CGC TCC TGG-3′	
*PGR*	NM_001003074	Forward: 5′-CGA GTC ATT ACC TCA GAA GAT TTG TTT-3′	113
		Reverse: 5′-CTT CCA TTG CCC TTT TAA AGA AGA-3′	
		TaqMan Probe: 5′-AAG CAT CAG GCT GTC ATT ATG GTG TCC TAA CTT-3′	
*ESR1 (ERα)*	XM533454	Forward: 5′-CCC ATG GAG GAG ACA AAC CA-3′	93
		Reverse: 5′-CCC TGC CTC GGT GAT ATA-3′	
		TaqMan Probe: 5′-CAC GGG CCC AAC TTC ATC ACA TTC C-3′	
*ESR2 (ERβ)*	XM861041	Forward: 5′-CCC AGC CCC TTC A-3′	78
		Reverse: 5′-AAT CAT ATG CAC GAG TTC CTT GTC-3′	
		TaqMan Probe: 5′-CCT CCA TGA TGA TGT CCC TGA CC-3′	
*PTGS2 (COX2)*	HQ110882	Forward: 5′-GGA GCA TAA CAG AGT GTG TGA TGT G-3′	87
		Reverse: 5′-AAG TAT TAG CCT GCT CGT CTG GAA T-3′	
		TaqMan Probe: 5′-CGC TCA TCA TCC CAT TCT GGG TGC-3′	
*AKR1C3 (PGFS)*	NM_001012344	Forward: 5′-AGG GCT TGC CAA GTC TAT TGG-3′	74
		Reverse: 5′-GCC TTG GCT TGC TCA GGA T-3′	
		TaqMan Probe: 5′-TCC AAC TTT AAC CGC AGG CAG CTG G-3′	
*PTGES*	NM_001122854	Forward: 5′-GTC CTG GCG CTG GTG AGT-3′	89
		Reverse: 5′-ATG ACA GCC ACC ACG TAC ATC-3′	
		TaqMan Probe: 5′-TCC CAG CCT TCC TGC TCT GCA GC-3′	
*PTGFR (FP)*	NM_001048097	Forward: 5′-ACC AGT CGA ACA TCC TTT GCA-3′	86
		Reverse: 5′-GGC CAT CAC ACT GCC TAG AAA-3′	
		TaqMan Probe: 5′-CAT GGT GTT CTC CGG TCT GTG CCC-3′	
*PTGER2 (EP2)*	AF075602	Forward: 5′-CAC CCT GCT GCT GCT TCT C-3′	78
		Reverse: 5′-CGG TGC ATG CGG ATG AG-3′	
		TaqMan Probe: 5′-TGC TCG CCT GCA ACT TTC AGC GTC-3′	
*PTGER4 (EP4)*	NM_001003054	Forward: 5′-AAA TCA GCA AAA ACC CAG ACT TG-3′	96
		Reverse: 5′-GCA CGG TCT TCC GCA GAA-3′	
		TaqMan Probe: 5′-ATC CGA ATT GCT GCT GTG AAC CCT ATC C-3′	
*SLCO2A1 (PGT)*	NM_001011558	Forward: 5′-TGC AGC ACT AGG AAT GCT GTT C-3′	116
		Reverse: 5′-GGG CGC AGA GAA TCA TGG A-3′	
		TaqMan Probe: 5′-TCT GCA AAC CAT TCC CCG CGT G-3′	

### Western blot analysis

NET-2 lysis buffer (50 mM Tris–HCl, PH 7.4, 300 mM NaCl, 0.05 % NP-40) containing 10 μl/ml protease inhibitor cocktail (Sigma-Aldrich Chemie) was used to prepare whole cell lysates for the immunoblotting analyses which were performed as previously described [33]. Briefly: protein homogenates (20–30 μg of total protein) were separated on 10 % polyacrylamide gel (Bio-Rad Laboratories GmbH, Munich, Germany) and transferred onto methanol-activated polyvinylidene difluoride (PVDF) membranes (Bio-Rad) and probed with specific antibodies. Due to the limited availability of canine-specific and/or cross reacting antibodies suitable for Western blot analysis, detection of decidualisation markers using this method was restricted to the following target proteins that are significantly upregulated during decidualisation, and for which Western blot-suitable antisera were available: COX2 (PTGS2; using monoclonal mouse anti-rat PTGS2 IgG, clone 33, BD Pharmingen, Heidelberg, DE; dilution 1:500), and PTGES and PGFS/AKR1C3 (both guinea pig polyclonal, affinity-purified anti-dog, Eurogentec S.A., Seraing, Belgium; dilutions 1:25 and 1:1000, respectively; [33, 34]). The PVDF membranes were reblotted with monoclonal mouse anti-human GAPDH IgG (sc-47724) from Santa Cruz Biotechnology, Inc., Santa Cruz, CA, USA, at a dilution of 1:1000. Secondary antibodies were: goat anti-mouse IgG (W402B) from Promega (dilution 1:15,000) and anti-guinea pig IgG conjugated HRP from Sigma-Aldrich (A5545; 1:15,000). Detection of signals was performed using SuperSignal West chemiluminescent substrate (Life Technologies Europe B.V., Zug, CH) according to the manufacturer’s protocol; visualization of signals was done using the ChemiDoc XRS+ System and Image Lab Software, both from Bio-Rad. The optical density of bands was assessed using ImageJ software. In order to test for the effects of time and treatment on COX2 (PTGS2) and PTGES expression, an unpaired two-tailed Student’s *t*-test was performed with the GraphPad 3.06 software program. Numerical data are presented as the mean ± standard deviation (SD). Representative Western blots are shown.

## Results

### Characterisation and *in vitro* decidualisation of isolated canine uterine stromal cells

**Fig. 1:**
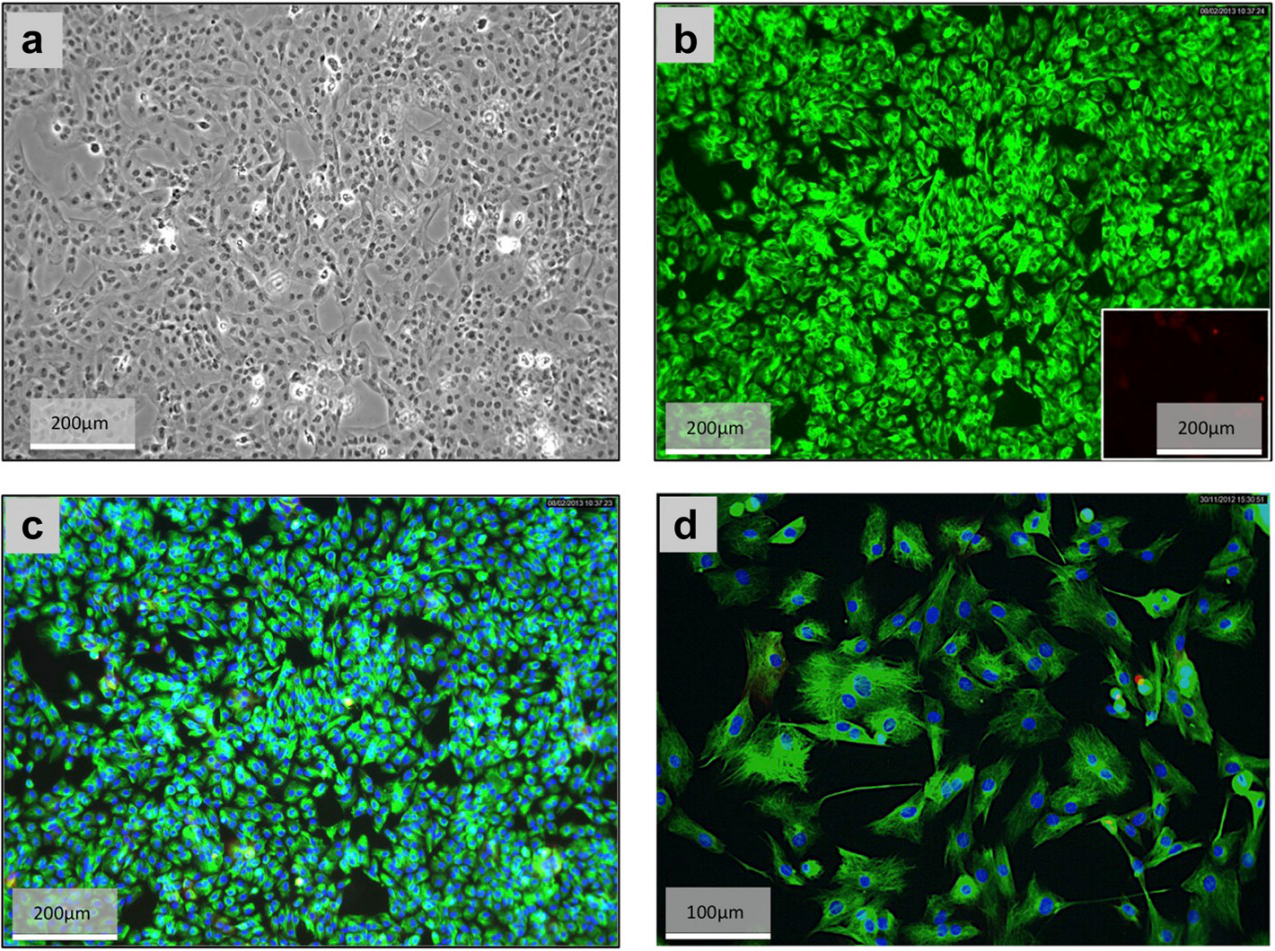
Canine primary uterine stromal cells. **a** A high confluence monolayer is presented. **b** Vimentin (green) and cytokeratin (red, inset in **b**) immunofluorescence was performed in order to confirm the mesenchymal origin of uterine stromal cells. There is no or only weak staining for cytokeratin visible indicating high purity of isolated stromal cells. **c** and **d** present merged pictures at lower and higher magnifications with cells stained for vimentin and cytokeratin; nuclear staining was achieved with 4′,6-Diamidino-2-phenylindole (DAPI)

**Fig. 2:**
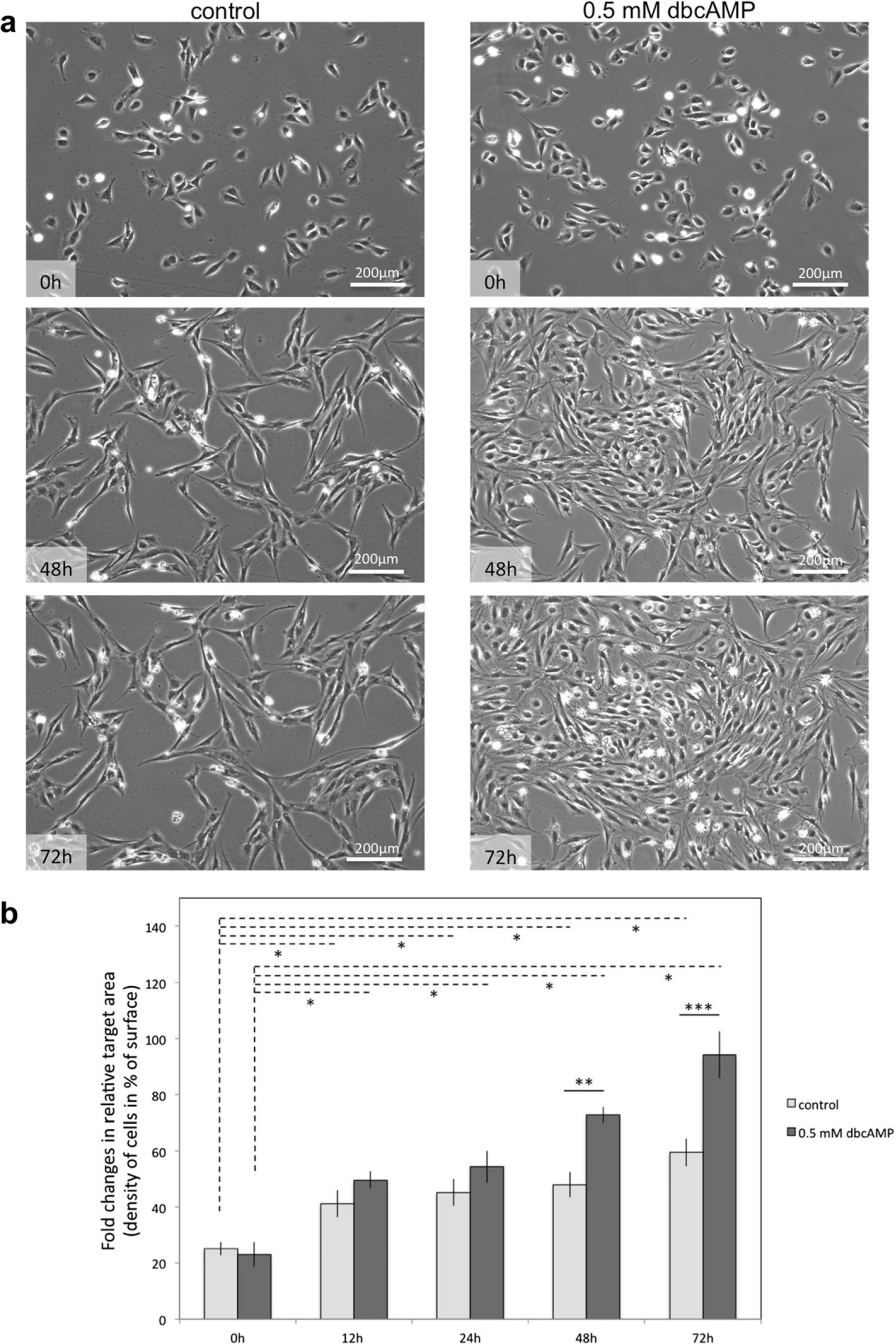
Morphological appearance of canine uterine stromal cells during *in vitro* decidualisation (**a**). Quantitation of cell density: percentage (%) of surface determined under live cell imaging conditions (**b**; average from three independent experiments). One-way ANOVA followed by Dunnett’s Multiple Comparison Test was applied to test the effects of time on cell density in all control or treated samples ((*) indicates *P* < 0.0001). Student’s *t-*test was applied to test the effect of treatment on cell density: (**) indicates *P* < 0.0002, (***) indicates *P* < 0.0007

Primary stromal cells were isolated from uteri of dogs collected during the early dioestrus phase using enzymatic dissociation and using the differential adhesion time. Nearly 100 % of cells stained positively for vimentin, thus verifying their mesenchymal origin (Fig. 1). Only sporadically were epithelial cell-derived cytokeratin signals detected. The morphological changes observed during *in vitro* decidualisation were consistent with the acquired characteristics of decidual cells. The shape of cells changed from elongated and spindle-shaped to rounded (Fig. 2a) when cells were incubated for 72 h with 0.5 mM dbcAMP. Morphological changes observed during *in vitro* decidualisation of canine stromal cells were captured using time-lapse microscopy and are presented in the supplemental material (Additional file 1). Simultaneously with the morphological changes, the *in vitro* decidualised cells revealed an accelerated proliferation rate as determined by quantification of cell densities in time-lapse derived pictures at the selected time points of 0, 12, 24, 48 and 72 h (Fig. 2a). Whereas both dbcAMP-stimulated and control cell densities increased significantly over time (*P* < 0.0001), the density of decidualised cells significantly exceeded that of control cells by 48 h and 72 h (*P* < 0.0002 and *p* < 0.0007, respectively) (Fig. 2b). Nearly 100 % confluence was observed in decidualised cells by 72 h.

**Fig. 3:**
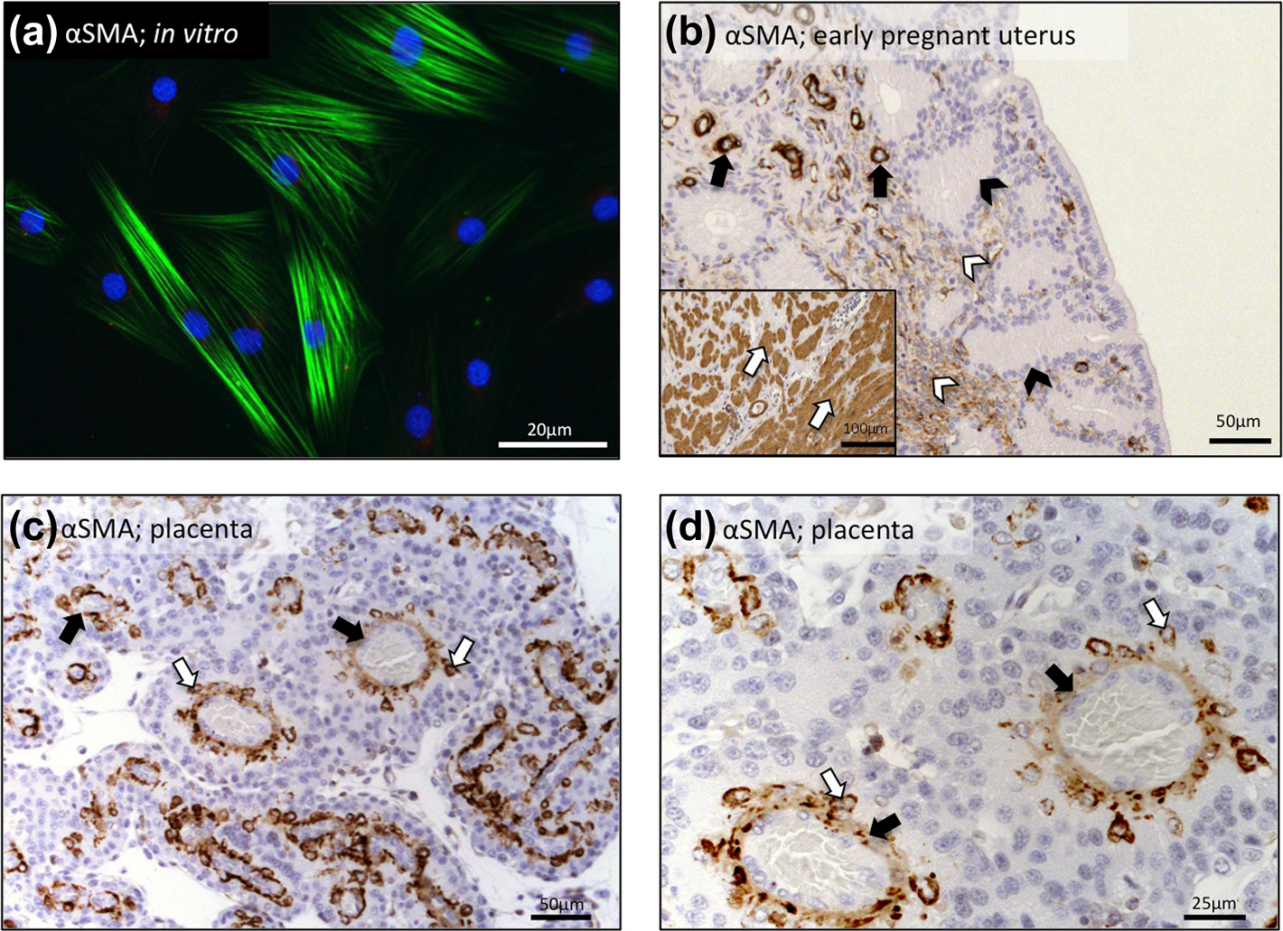
Immunostaining against smooth muscle alpha actin (αSMA). **a** Decidualised canine stromal cells expressing αSMA (green); blue nuclear staining was achieved by DAPI. **b** Immunohistochemical localization of αSMA in canine pre-implantation uterus (solid arrows = blood vessels; open arrows = smooth muscle cells of myometrium, open arrowheads = uterine stromal cells, solid arrowheads = uterine glands). **c**, **d** Immunohistochemical localization of αSMA in canine mid-term placenta (solid arrows = large maternal vessels; open arrows = decidual cells)

Decidualised canine stromal cells displayed strong immunocytochemical signals for αSMA (Fig. 3a). Its expression was compared with the expression and localization of αSMA in the pre-implantation canine uterus and in mid-term placenta. Prior to implantation, strong signals were localized in smooth muscle cells of the myometrium and tunica media and basement membrane of blood vessels, while weaker staining was observed in endometrial stromal cells (Fig. 3b). In addition to the vascular staining, within the placenta strong signals were localized in maternal decidual cells (Fig. 3c,d).

### Subcellular morphological characterisation of canine decidual cells during *in vitro* decidualisation

**Fig. 4:**
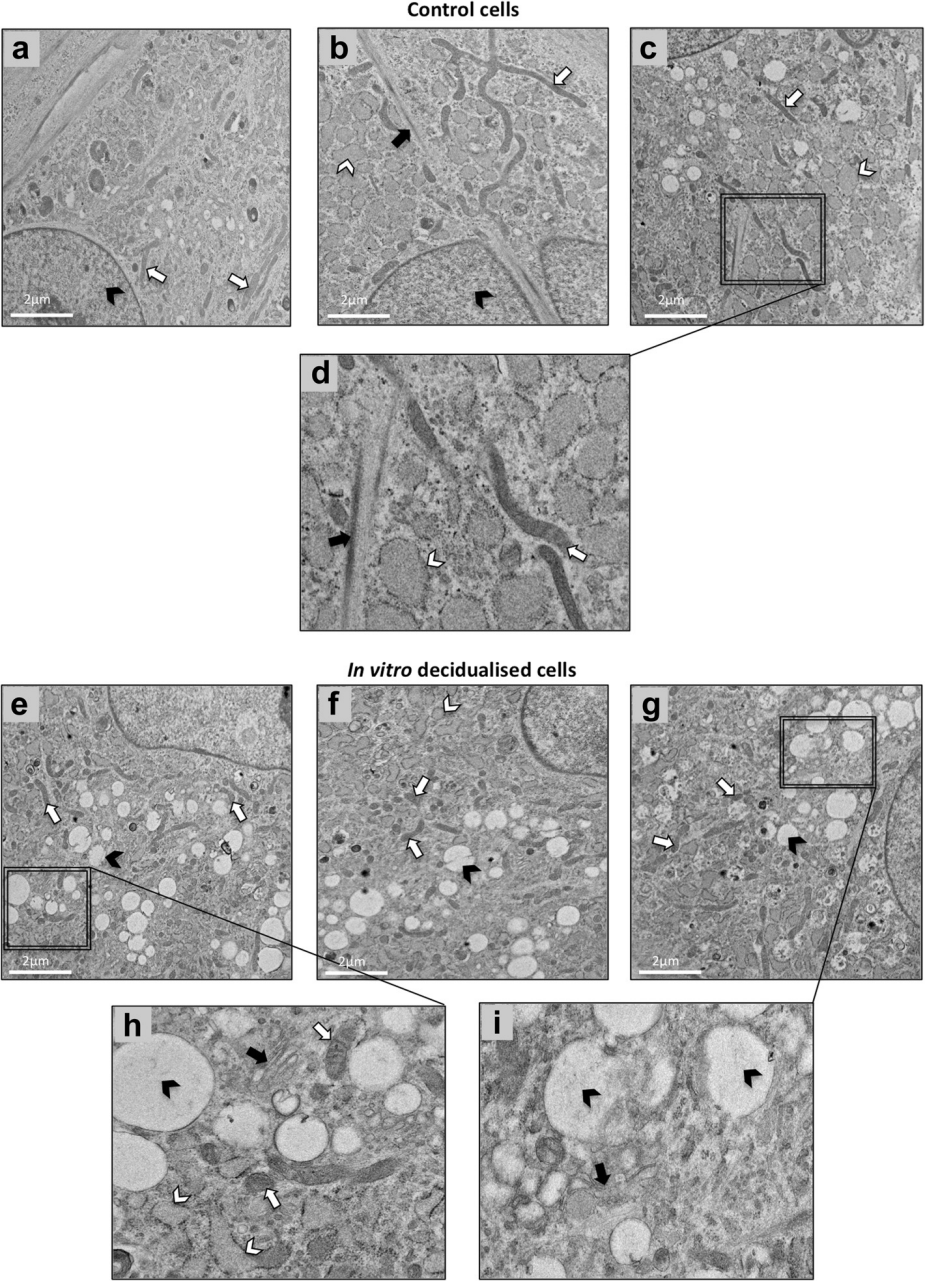
Transmission electron microscopy (TEM) analysis of canine uterine primary stromal cells during *in vitro* decidualisation. **a**-**d** control cells at 72 h of culture (solid arrowheads = nucleus, open arrows = mitochondria, solid arrows = microfilaments, open arrowheads = cisternae of rough endoplasmic reticulum). **e**-**i** *in vitro* decidualised cells, after 72 h treatment with 0.5 mM dbcAMP (solid arrowheads = large secretory vesicles, open arrows = mitochondria, solid arrows = Golgi apparatus, open arrowheads = cisternae of rough endoplasmic reticulum)

**Fig. 5:**
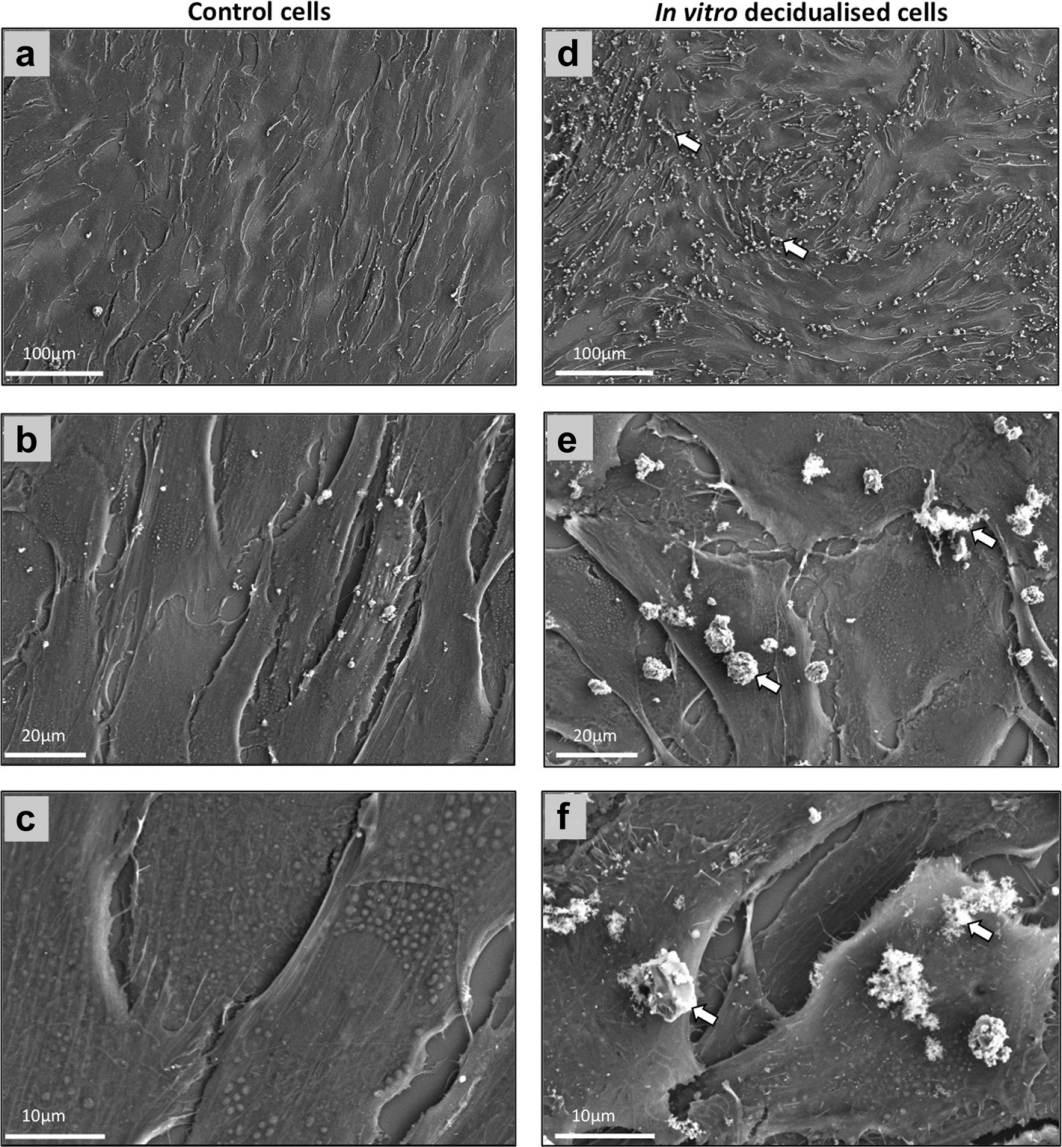
Scanning electron microscopy (SEM) imaging of canine uterine primary stromal cells during *in vitro* decidualisation. **a**-**c** control cells at 72 h of culture, (**d**-**f**) *in vitro* decidualised cells, after 72 h treatment with 0.5 mM dbcAMP. Increased secretory activity of decidualised cells is visible on cell surface, as characterized by larger amounts of secreted deposits (open arrows in **d**-**f**)

Transmission electron microscopic analysis (TEM) of control stromal cells revealed the presence of long, often branched mitochondria, frequently visible microfilaments and abundant cisternae of rough endoplasmic reticulum (Fig. 4a-d). Cells undergoing *in vitro* decidualisation were characterized by higher abundance of Golgi apparatus which is responsible for protein sorting, modification and packaging for secretion, and consequently, greater amounts of secretory vesicles (Fig. 4e-i). These cells also displayed higher numbers of small, round-shaped mitochondria. As revealed by scanning electron microscopic analysis (SEM), the increased secretory activity of decidualised cells was also clearly visible on cell surfaces, displaying higher amounts of secreted deposits than in non-treated controls (Fig. 5a-f).

### Expression of selected genes in canine uterine primary stromal cells during *in vitro* decidualisation

**Fig. 6:**
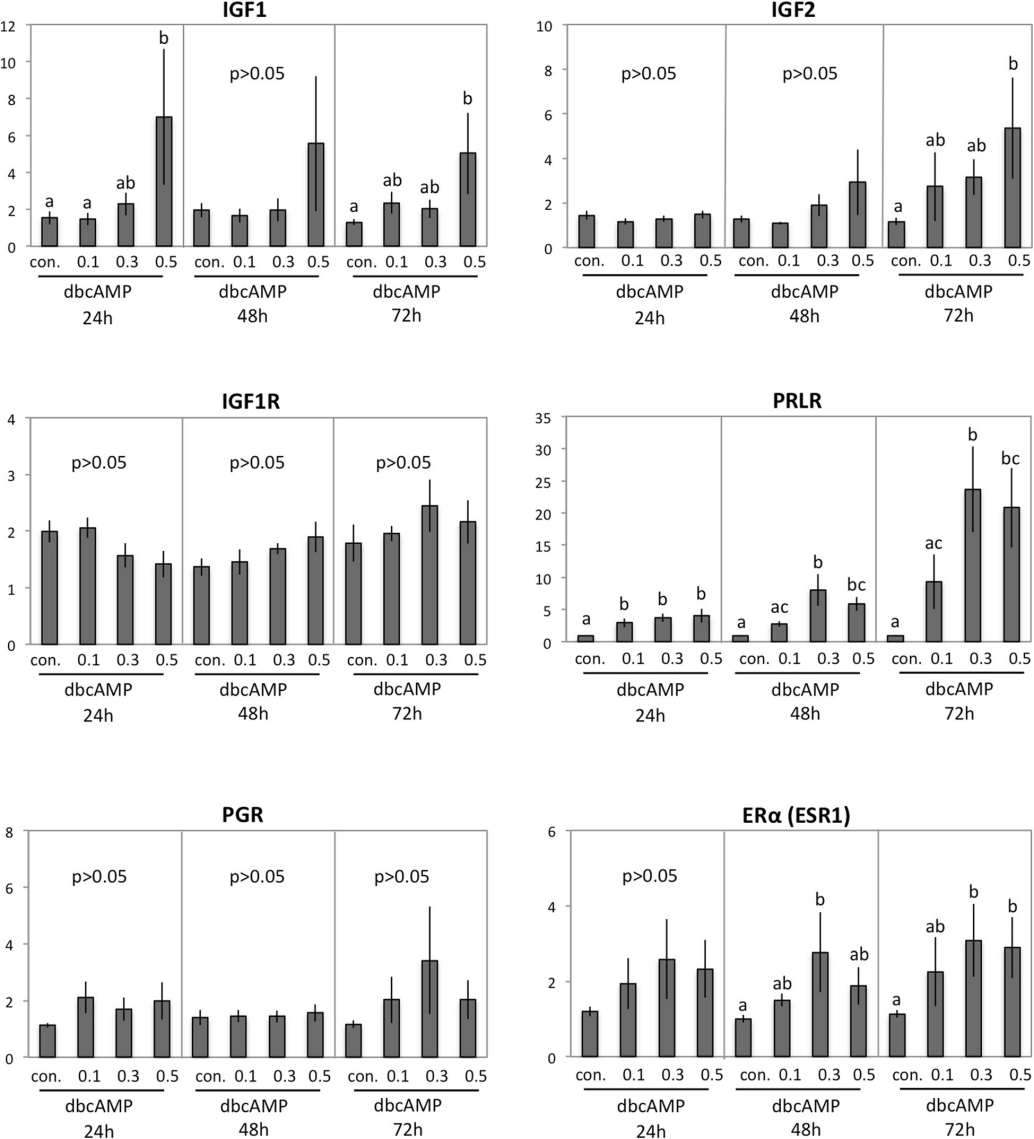
Expression of insulin-like growth factor 1 (IGF1), IGF2, IGF-receptor 1 (IGF1R), prolactin receptor (PRLR), progesterone receptor (PGR) and oestrogen receptor alpha (ERα, ESR1), as determined by Real Time (TaqMan) PCR. Canine primary stromal cells were cultured for 24, 48 and 72 h in the presence of increasing dbcAMP concentrations. One-way ANOVA (24 h in IGF1 *P* < 0.01, 72 h in IGF1 *P* < 0.02; 72 h in IGF2 *P* < 0.03; 24 h in PRLR *P* < 0.0003, 48 h in PRLR *P* < 0.0001, 72 h in PRLR *P* < 0.001; 48 h in ERα *P* < 0.02, 72 h in ERα *P* < 0.004), followed by the Tukey-Kramer Multiple Comparison Test was applied; all samples were compared against the non-treated control in each group. Different letters indicate *P* < 0.05. Numerical data are presented as the mean ± standard deviation (SD)

Table 1 presents a list of several selected genes, the expression of which was investigated at the mRNA level during dbcAMP-induced *in vitro* decidualisation of canine uterine stromal cells. Non-stimulated cells served as controls over the 72 h time course of the study. Whereas the expression of IGF1R did not change significantly during the period of *in vitro* decidualisation, IGF1 was already strongly upregulated (*P* < 0.05) after 24 h in response to 0.5 mM dbcAMP, while the expression of IGF2 was significantly elevated at 72 h (*p* < 0.05) (Fig. 6). The expression of PRLR increased significantly over controls within 24 h, responding to low and high dbcAMP concentrations (*P* < 0.05, *P* < 0.01 and *P* < 0.001 for 0.1 mM, 0.3 mM and 0.5 mM dbcAMP, respectively). This effect was also apparent in cells incubated for 48 h (*P* < 0.001 and *P* < 0.01 for 0.3 mM and 0.5 mM dbcAMP, respectively). The highest PRLR response was noted at 72 h in response to 0.3 mM and 0.5 mM dbcAMP, *P* < 0.001 and *P* < 0.01, respectively (Fig. 6). In contrast, the expression of PRL mRNA remained low and frequently below the detection limit both in control and stimulated cells throughout the experimental time-course (not shown).

The evaluation of steroid hormone receptor expression revealed significantly (*P* < 0.05) altered expression of ERα (ESR1) during *in vitro* decidualisation, presenting elevated mRNA levels after 48 h and 72 h in cells stimulated with 0.3 mM or 0.5 mM dbcAMP. The expression of PGR did not change significantly in decidualised cells (P > 0.05) (Fig. 6). Similarly, as for PRL, the expression of ERβ (ESR2) mRNA remained mainly below the detection limit in all samples throughout the experiment (not shown).

**Fig. 7:**
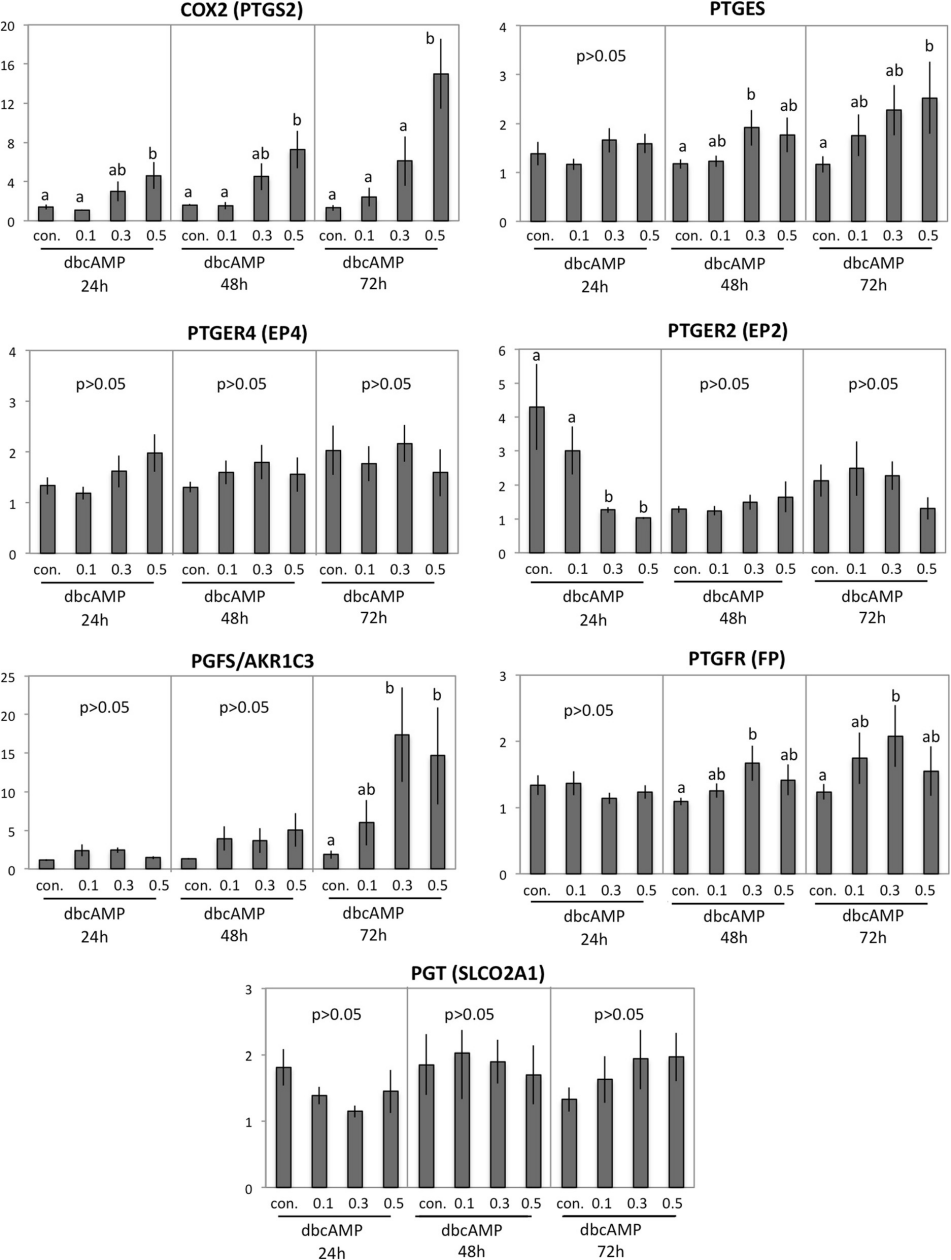
Expression of cyclooxygenase 2 (COX2, PTGS2), PGE2-synthase (PGES, PTGES), PGE2 receptors: EP4 (PTGER4) and EP2 (PTGER2), PGF2α-synthase (PGFS/AKR1C3), PGF2α receptor (PTGFR, FP) and PG-transporter (PGT, SLCO2A1), as determined by Real Time (TaqMan) PCR. Canine primary stromal cells were cultured for 24, 48 and 72 h in the presence of increasing dbcAMP concentrations. One-way ANOVA was applied (24 h in COX2 *P* < 0.005, 48 h in COX2 *P* < 0.01, 72 h in COX2 *P* < 0.0001; 48 h in PGES *P* < 0.01, 72 h in PGES < 0.03; 24 h in EP2 *P* < 0.0001, 72 h in PGFS P < 0.03, 48 h in FP *P* < 0.03, 72 h in FP *P* < 0.01), followed by the Tukey-Kramer Multiple Comparison Test; all samples were compared against the non-treated control in each group. Different letters indicate *P* < 0.05. Numerical data are presented as the mean ± standard deviation (SD)

Among the major members of the prostaglandin system, the expression of COX2 (PTGS2) displayed an expression pattern similar to that observed for IGF2 (Fig. 7). It was, however, already significantly upregulated 24 h after stimulation in response to 0.5 mM dbcAMP (*P* < 0.01). As for IGF2, its highest expression above controls was noted at 72 h (*P* < 0.001) in response to 0.5 mM dbcAMP. Similarly, the expression of PGFS/AKR1C3 resembled that of IGF2 and was significantly increased at 72 h in response to both 0.3 mM and 0.5 mM dbcAMP compared to the respective controls (*P* < 0.05). The PGF2α receptor PTGFR (FP) and PTGES presented similar expression levels (Fig. 7). Thus, while displaying greater variations, their expression levels were increased (*P* < 0.05) at 48 h after stimulation. Especially for PTGES, this effect was obvious at 72 h in response to the highest dbcAMP dosage (*P* < 0.05). Contrasting with this was the expression of the respective PGE2 receptors, which either did not change significantly (P > 0.05) for EP4 (PTGER4) or decreased significantly for EP2 (PTGER2), both in response to dbcAMP within the first 24 h of incubation (*P* > 0.001) when compared with the respective control, as well as in all controls over the entire time course (*P* < 0.001 for 48 h and *P* < 0.01 for 72 h, respectively).

**Fig. 8:**
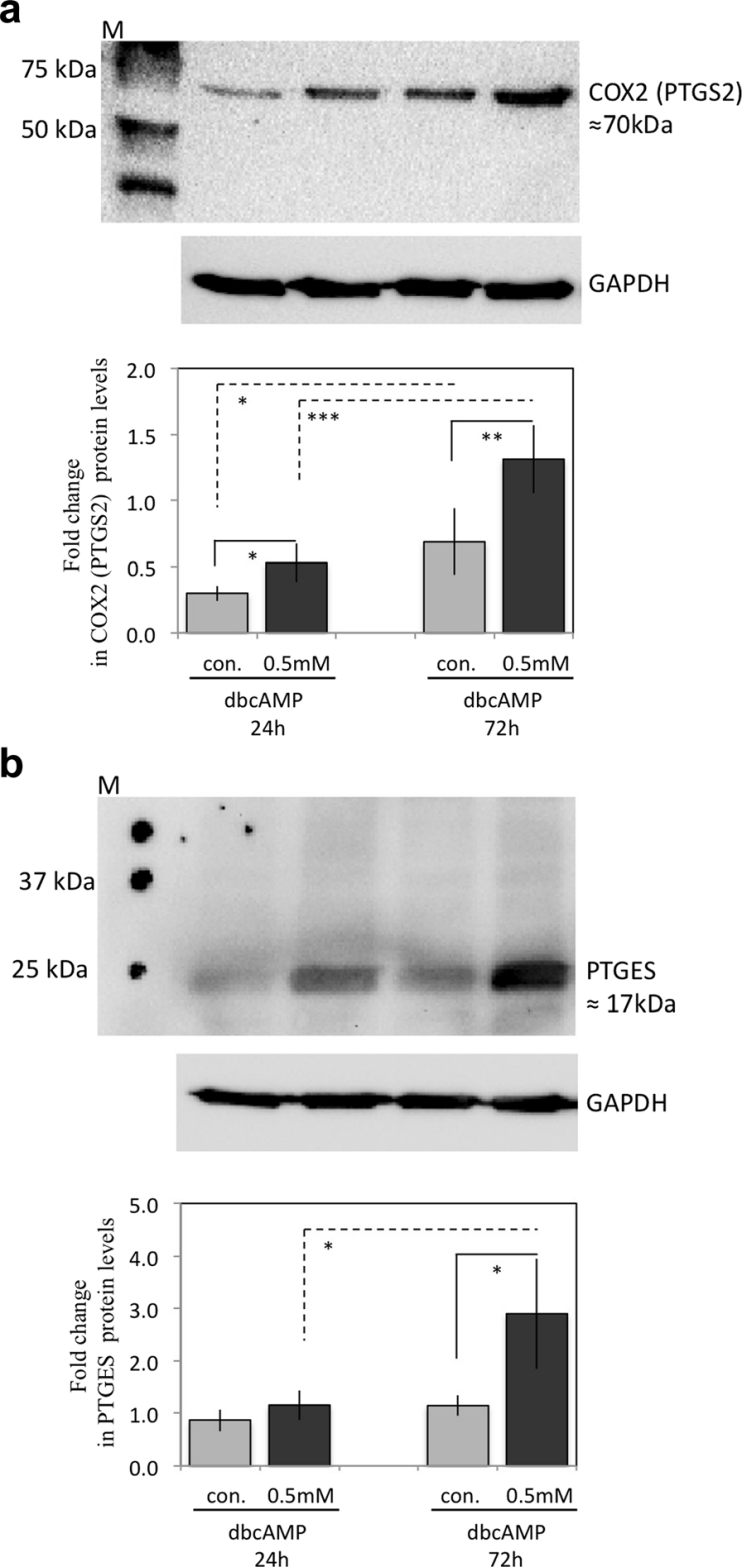
Expression of cyclooxygenase 2 (COX2, PTGS2) and PGE2-synthase (PGES, PTGES) as determined by Western blot analysis. Following stimulation, cells were collected and homogenized and 20 μg of the lysate was used for Western blots. The averaged standardized optical density (SOD) is shown. GAPDH was used for loading control. The cell culture experiments were performed independently at least three times using cells isolated from different animals. Representative Western blots are shown. Student’s *t-*test was applied to test the effect of treatment on protein expression: in **a** (*) indicates *P* < 0.01, (**) indicates *P* < 0.004, (***) indicates *P* < 0.0001; in **b** (*) indicates *P* < 0.008

Similarly, as for EP4, the expression of PGT (SLCO2A1) remained unaffected (*P* > 0.05) during decidualisation. At the protein level*,* PTGS2 and PTGES expression profiles matched their respective mRNA levels, as shown in Fig. 8 for 24 h and 72 h of stimulation. Thus, whereas COX2 (PTGS2) expression already increased significantly (*P* < 0.01) at 24 h, both PTGS2 and PTGES were strongly upregulated at 72 h (*P* < 0.004 and *p* < 0.007, respectively), compared with their respective controls. Although the canine-specific anti-PGFS/AKR1C3 antibody previously tested positively on canine uteri and placenta [33], within the current study the expression of PGFS/AKR1C3 protein remained below the detection limit (not shown).

## Discussion

Decidualisation is a complex process, during which species displaying invasive types of placentation develop highly specialized maternal stroma-derived decidual cells. Their roles are in facilitating implantation and supporting embryo growth, *e.g.,* by secreting hormones, such as prolactin or placental lactogen, or cytokines involved in immunological protection of the foetus [35–37]. None of these functions, however, are known for the dog.

Although devoid of an anti-luteolytic mechanism that serves in other domestic animal species to prevent luteolysis, the canine uterus was recently shown to respond to the presence of pre-implantation, free-floating embryos by activating the expression of markers of ongoing, early decidualisation [15]. Following implantation and placentation, canine decidual cells eventually develop. These are the most important sensors of luteal P4 at the foeto-maternal interface in the dog [20]. Until now, the process of decidual cell formation in the dog was unknown, and neither was there an available cellular model allowing for detailed *in vitro* studies. Therefore, the present study investigated the basic capability of canine uterine stromal cells isolated from bitches during early dioestrus to decidualise *in vitro*. The dbcAMP-based protocol was chosen to compensate for our limited access to the tissue material which did not allow more detailed studies utilizing, *e.g*., ovarian steroids. Nevertheless, dbcAMP has proved to be a potent stimulus mediating P4-driven decidualisation [23, 38]. Among other cAMP inducers capable of initializing decidualisation of uterine stromal cells are relaxin and gonadotropins [39, 40]. In line with this, responding to dbcAMP, canine stromal cells acquired the characteristics of decidual cells, reaching nearly 100 % confluency at 72 h. They changed morphologically, became rounded, exhibited accelerated proliferation rates and increased their metabolic activity, as concluded from the increased incidence of small mitochondria, elevated numbers of secretory follicles and increased deposits of extracellular matrix observed under scanning electron microscope. The composition and function of the matrix components secreted by these cells in dogs needs to be determined. As a further sign of successful *in vitro* decidualisation, strong expression of αSMA was observed in these decidualised cells. Its expression is a typical sign of decidualisation known from other species including humans and is also an important marker used for detection of endometriotic lesions in women [41]. During the pre-implantation stage of pregnancy, the expression of αSMA in canine uterine stromal cells was only weak, indicating an early stage of uterine differentiation. Following placentation, it was localized and abundantly expressed in decidual cells of the *placenta materna*, which conformed to our *in vitro* findings.

At the biochemical and functional levels, the process of canine decidualisation still remains to be elucidated. In the present study, in order to validate the newly established *in vitro* model, the presence of several factors was investigated, the expression of which was recently described in early pregnant canine uterus and/or placenta [15, 20, 33, 34]. Some of them, such as IGF1 and IGF2 are among the most prominent decidualisation markers in other species [42, 43]. Others, like PGs, even if *per se* are not decidual cells markers, belong to strong inducers of the decidualisation process [23].

Thus, acting predominantly at the level of their common receptor IGFR1, both IGFs are mitogenic factors [42, 43]. They drive embryonic development and are important determinants of fetal and placental growth (reviewed in [44]). Their expression during *in vitro* decidualisation of canine stromal cells, as well as the expression of other factors investigated in the present study, was found to closely resemble their expression patterns evoked in early pregnant uteri in response to free-floating embryos [15]. Hence, both IGF1 and IGF2 responded positively to the applied decidualisation protocol. Whereas IGF1 turned out to be a quick responder displaying a significant dbcAMP dose-dependent response within 24 h, IGF2 responded in a time-dependent manner with its highest expression observed only after 72 h. Similar effects on IGF2 expression, becoming significant after 3 days of *in vitro* decidualisation, were observed in human endometrial stromal cells [38]. In contrast to human and rodent models, however, in which PRL is one of the strongest decidualisation markers [38, 45, 46] involved in regulating endometrial secretory activity [47], canine decidualised cells exhibited very low expression of PRL. This, together with the concomitantly highly upregulated expression of PRLR observed in these cells, confirms our previous findings on the low expression of PRL in canine uteri which is frequently below detection limits [15], and points towards species-specific regulatory mechanisms. As part of these, it appears plausible that the strongly elevated levels of PRLR could compensate for low PRL expression, locally increasing its relative biological availability. This could also apply to the expression of ERα, which in contrast to ERβ was strongly elevated during decidualisation, possibly compensating for the low availability of circulating oestradiol of luteal origin during canine gestation. This, indeed, strongly resembles the situation observed *in vivo* where higher ERα mRNA and protein levels were observed in early pregnant canine uteri compared to non-pregnant controls [15]. In this regard, interestingly, in naturally oestrogenized dogs, no additional supplementation with oestrogens is needed for establishment and maintenance of pregnancy. This was clearly shown by Concannon [48, 49] in experiments with dogs ovariectomized early in gestation (day 14 after the preovulatory LH surge), in which pregnancy was maintained only due to exogenous supplementation with P4. Although unaltered during *in vitro* and early *in vivo* decidualisation, the expression of PGR in canine decidual cells is a *sine qua non* condition for successful establishment and maintenance of canine pregnancy by preventing the premature luteolytic cascade [15, 20].

In human decidua, IGF1 was shown to be involved in regulation of arachidonic acid secretion, which is a common precursor for PG synthesis [50]. Similarly, in our experiments the *in vitro* expression of PG synthases closely paralleled the expression of IGFs. The functional interrelationship between these entities remains to be elucidated for the dog.

The versatile functions of PGs include the cAMP/PKA pathway-mediated stimulatory effects of PGE2 in further enhancing P4 and cAMP-induced decidualisation in humans [23]. Also, immunosuppressive but embryo-protective effects of human decidua-derived PGE2 were proposed [51]. Furthermore, in addition to the species-specific function of oestrogens, both, embryo- and luteo-protective roles of endometrial PGE2 have been proposed for pigs (see review [52]). Since all of the above functions could also apply to the dog, the modulatory effect of decidualisation on the expression of PG family members in canine stromal cells is noteworthy. PGs, and especially PGE2, are among the most important luteotrophic factors in the dog [27, 53–55]. Together with IGF2, abundant expression of PTGES was observed in hatched canine pre-implantation embryos and the pre-implantation uterus [15]. Interestingly, during placentation PGs are more strongly represented in the foetal compartments [20, 33, 34]. However, the increased expression shown herein of COX2 and PTGES transcripts, but not those of PGFS/AKR1C3, was clearly mirrored at the protein level. Thus, in addition to the foetal trophoblast, decidual cells appear to be an important potential source of canine placental PGs. By expressing the respective PG receptors and PGT (SLCO2A1), they are most likely involved in paracrine and autocrine local regulatory mechanisms.

## Conclusion

Taking into account the indispensable function of decidual cells in ensuring proper uterine secretory activity, as well as their roles during the establishment and maintenance of canine pregnancy, herein a valuable *in vitro* model was established showing the basic capability of canine uterine stromal cells to decidualise *in vitro*. The morphological and biochemical characteristics of decidual cells were acquired during this process, providing evidence that canine decidualisation can be effectively modeled *in vitro*. Nevertheless, further investigations are needed to dissect the underlying molecular and endocrine mechanisms involved. In particular, the role of upstream operating regulatory mechanisms responsible for initializing the cAMP-dependent decidualisation cascade, needs to be addressed.

## Additional file

Additional file 1:
**Time-lapse videos of** ***in vitro*** **decidualising canine uterine stromal cells compared with untreated controls.** Both representative videos were taken simultaneously during one experiment over 72 h (movies are displayed at 100× magnification).
